# Dicarbonyl Stress and S-Glutathionylation in Cerebrovascular Diseases: A Focus on Cerebral Cavernous Malformations

**DOI:** 10.3390/antiox9020124

**Published:** 2020-02-01

**Authors:** Cinzia Antognelli, Andrea Perrelli, Tatiana Armeni, Vincenzo Nicola Talesa, Saverio Francesco Retta

**Affiliations:** 1 Department of Experimental Medicine, University of Perugia, 06132 Perugia, Italy; vincenzo.talesa@unipg.it; 2 Department of Clinical and Biological Sciences, University of Torino, Orbassano, 10043 Torino, Italy; andrea.perrelli@unito.it; 3 Department of Clinical Sciences, Polytechnic University of Marche, 60131 Ancona, Italy; t.armeni@univpm.it

**Keywords:** cerebrovascular disease, cerebral cavernous malformations, dicarbonyl stress, glyoxalase 1, methylglyoxal, advanced glycation end products, glutathione, S-glutathionylation, oxidative stress

## Abstract

Dicarbonyl stress is a dysfunctional state consisting in the abnormal accumulation of reactive α-oxaldehydes leading to increased protein modification. In cells, post-translational changes can also occur through S-glutathionylation, a highly conserved oxidative post-translational modification consisting of the formation of a mixed disulfide between glutathione and a protein cysteine residue. This review recapitulates the main findings supporting a role for dicarbonyl stress and S-glutathionylation in the pathogenesis of cerebrovascular diseases, with specific emphasis on cerebral cavernous malformations (CCM), a vascular disease of proven genetic origin that may give rise to various clinical signs and symptoms at any age, including recurrent headaches, seizures, focal neurological deficits, and intracerebral hemorrhage. A possible interplay between dicarbonyl stress and S-glutathionylation in CCM is also discussed.

## 1. Introduction

Cerebrovascular diseases (CeVDs) are a leading cause of serious long-term disability, deeply impacting prognosis and health-related quality of life of patients, and the second-leading cause of death worldwide [1]. They constitute a subset of cardiovascular diseases (CVDs), consisting of a spectrum of different subclinical and clinical disorders that affect the blood vessels of the brain and the cerebral circulation, including stroke, aneurysms, and vascular malformations. Although CeVDs are diverse, complex, and multifactorial diseases caused by multiple interactions between vascular, environmental, and genetic factors, including distinct gene-gene and gene-environment interactions [2,3,4], there is compelling evidence that the underlying pathogenetic mechanisms are often similar, mainly concerning endothelial dysfunction caused by oxidative stress (OS) and/or inflammation [5,6,7]. In fact, the endothelium lining in the cardiovascular system is not merely a physical barrier between blood and the vascular wall but rather a real “organ” endowed with autocrine and paracrine properties fundamental for maintaining vascular homeostasis, which can be instead compromised by stressful conditions [8]. Consistently, most of the potentially modifiable environmental risk factors for CeVDs, including smoking, hypertension, dyslipidemia, obesity, metabolic syndrome, and type 2 diabetes, act primarily by promoting endothelial dysfunction through the induction of OS and inflammatory events [7]. In particular, OS, which is generated by an imbalance of the physiological equilibrium between the generation of reactive oxygen species (ROS) and the capacity of cellular antioxidant mechanisms to promptly and effectively detoxify the reactive intermediates or repair the resulting damage, was plainly shown to promote endothelial dysfunction and cerebrovascular diseases by affecting multiple redox-sensitive molecular mechanisms [5,6,9]. ROS can be produced by all vascular cell types, including endothelial and periendothelial cells and migrating inflammatory cells [10,11,12]. At the cellular level, ROS are generated by multiple sources, including the mitochondrial electron-transport chain (mETC), cytochrome P450 monooxygenases (CYP), nitric oxide synthases (NOS), cyclooxygenases (COX), lipoxygenases (LOX), xanthine oxidase (XOD), and the nicotinamide adenine dinucleotide phosphate (NADPH) oxidase (Nox) family of enzymes [12,13]. Above certain threshold levels, ROS can promote endothelial dysfunction either directly or by depleting the bioavailability of nitric oxide (NO), a major vasodilator factor responsible for the preservation of vascular integrity and vasomotor function [8]. In particular, several studies have shown that overproduction of distinct ROS, including O_2_•^–^, HO•^–^, and H_2_O_2_ can elicit endothelial barrier dysfunction by affecting the cytoskeletal architecture of the endothelial monolayer. For example, specific oxidants, including H_2_O_2_, were shown to either increase the phosphorylation of myosin light chain kinase [14], reduce the ability of Ca^2+^-bound G-actin to polymerize, or destroy the cortical actin rim, which is fundamental for maintaining endothelial barrier integrity [15]. In addition, ROS can induce endothelial dysfunction and related pro-atherosclerotic processes by generating oxidized LDL (OxLDL). In fact, OxLDL infiltrate vascular walls and induce the production of cytokines, which, in turn, promote vascular inflammation [16,17]. Furthermore, OxLDL are able to exert direct cytotoxic damage on endothelial cells of the cerebral vascular tree [18], to induce the release of vascular matrix metalloproteinases (MMPs) that trigger vessel destabilization [19], and to promote thrombocyte adhesion to the vascular wall [20]. In turn, these events may alter the integrity and permeability of the blood-brain barrier (BBB) [21], a vital semipermeable boundary between the central nervous system (CNS) and the peripheral circulation that regulates the selective transport of molecules and protects against external toxins and pathogens, thus causing cerebrovascular dysfunctions and diseases [22].

Counteracting OS is fundamental to limit OS-associated cellular damaging effects and is mainly based on antioxidants, molecules able to quench ROS. The neurovascular unit (NVU), which consists of highly specialized brain microvascular endothelial cells (BMECs), pericytes, astrocytes, and neurons, is endowed with fundamental nonenzymatic and enzymatic antioxidant defenses, including the tripeptide glutathione (GSH) and major antioxidant enzymes, such as glutathione peroxidase (GPX), glutathione reductase (GSR), peroxiredoxin (PRDX), superoxide dismutase (SOD), and catalase (CAT) [23]. In turn, these antioxidant defenses are modulated and implemented by nuclear factor erythroid 2-related factor (Nrf2), a master antioxidant transcription factor that is activated in response to OS and induces the expression of an array of detoxifying and antioxidant defense genes by binding to their antioxidant response element (ARE), thus regulating cellular responses to oxidative insults [24]. In particular, GSH, a thiol-containing tripeptide consisting of glutamic acid attached via its side chain to the N-terminus of cysteinyl-glycine (Glu-Cys-Gly, γ-glutamyl-cysteinyl-glycine), is the most important nonenzymatic antioxidant synthesized in cells and plays a crucial role in the preservation of BBB integrity by facilitating cell protection against oxidative damage, either directly or indirectly [23,25]. Indeed, whereas it is clearly established that GSH is a fundamental low molecular weight hydrophilic antioxidant [26], growing evidence indicates that the impairment of glutathione homeostasis, including an abnormal decrease in the redox ratio between its reduced (GSH) and oxidised (GSSG) forms and the consequent shift toward more oxidizing intracellular redox conditions, may contribute to endothelial dysfunction through the process of S-glutathionylation, a redox-sensitive oxidative post-translational modification (OPTM) of reactive Cys in target proteins [27,28,29,30,31,32].

Recent evidence strongly suggests that also dicarbonyl stress, a pathological condition occurring when dicarbonyl metabolites accumulate inside cells as a consequence of their increased production and/or decreased detoxification, contributes to both micro-and macro-vascular dysfunctions [33,34]. Among dicarbonyl metabolites, methylglyoxal (MG) is the most reactive and with the highest endogenous flux [34]. In particular, MG can compromise physiological angiogenesis by leading to the formation of aberrant capillaries through the upregulation of vascular endothelial growth factor receptor 2 (VEGFR2) [35], impair viability, migration, and tube formation of endothelial cells [36,37], induce endothelial cell apoptosis and increase vessel permeability [38]. Glyoxalase 1 (Glo1), a cytoplasmic GSH-dependent enzyme, provides the primary defense against dicarbonyl stress by catalyzing the metabolism of MG [34], thus contributing to protect from MG-induced vascular cell dysfunction [34].

This narrative review summarizes the latest evidence supporting a role for dicarbonyl stress and S-glutathionylation in the pathogenesis of CeVD, with a focus on cerebral cavernous malformations (CCM). A possible interplay between dicarbonyl stress and S-glutathionylation in CCM disease pathogenesis is also discussed.

## 2. Cerebral Cavernous Malformations (CCM)

Cerebral cavernous malformations (CCM) are vascular malformations consisting of closely clustered, abnormally dilated, and leaky capillary channels (caverns) lined by a thin endothelium, which affect 0.3–0.5% of the population [6]. Although CCM lesions can occur anywhere in the body, they are more often symptomatic only when they are present in brain and spinal cord, where they represent 5–15% of all vascular malformations. In brain, CCM lesions can be single or multiple and may stay clinically silent for a lifetime or unpredictably give rise to different clinical symptoms, such as headaches, neurological deficits, seizures, stroke, and intra-cerebral hemorrhage [6]. This cerebrovascular disease is of proven genetic origin, having been associated with loss-of-function mutations of three genes, *CCM1*/*KRIT1*, *CCM2,* and *CCM3*/*PDCD10*, and may arise sporadically or is inherited as an autosomal dominant condition with incomplete penetrance and highly variable expressivity [39]. Increasing evidence in conditional knockout mouse models has demonstrated that the loss-of-function of *CCM* genes is not sufficient to induce CCM disease onset and progression, suggesting the necessary contribution of additional factors and determinants of disease emergence other that disease-predisposing *CCM* gene mutations, including stress events that may occur in the endothelium microenvironment and interindividual variability in stress responses [2,3,6]. According to this model, a mutation in one allele of a *CCM* gene would make endothelial cells more susceptible to local stressful events, eventually leading to endothelial cell dysfunction and development of CCM lesions. This possibility would be consistent with the presence and dynamic nature of multiple CCM lesions in familial cases and could also account for the incomplete clinical penetrance and highly variable expressivity and delayed, age-dependent onset of the disease among family members sharing the same genetic mutations. Of the three known *CCM* genes, *KRIT1* (*Krev interaction trapped 1*) appears to be the most significant, as its loss-of-function mutations account for at least 50% of familial cases. Accumulated findings demonstrate that KRIT1 plays a major role in fundamental redox-sensitive mechanisms that regulate cellular homeostasis and defenses against OS, including transcriptional pathways and autophagy, raising the possibility that KRIT1 loss-of-function has pleiotropic effects on multiple redox-dependent cellular structures and functions [40,41]. Accordingly, the important role of altered redox homeostasis and OS in CCM disease pathogenesis, originally suggested by a seminal paper of Goitre et al. in 2010 [42], has subsequently been confirmed and extended, pointing to a novel unifying mechanistic scenario that accommodates all the different molecular pathways so far associated with CCM disease pathogenesis [2,3,6,40,41,43,44,45,46,47,48,49,50,51,52]. In particular, recent evidence has pointed to abnormal Nrf2-mediated adaptive responses, dicarbonyl stress, and S-glutathionylation as novel emerging redox-sensitive mechanisms underpinning the pathogenesis of CCM disease [48,49,51]. On the other hand, these original findings have suggested novel pharmacological strategies to counteract CCM disease onset and severity. Among them, there is the potential modulation of the crosstalk between autophagy and redox homeostasis and signaling with multitarget compounds endowed with antioxidant and anti-inflammatory properties. Accordingly, this strategy resulted effectively in rescuing major pathological phenotypes in cellular and animal models of CCM disease [6,41,45,46,47,50,51,53,54].

## 3. Dicarbonyl Stress: An Imbalance Between Accumulation of Methylglyoxal and Functionality of Glyoxalases

Dicarbonyl stress occurs as a consequence of enhanced production and/or decreased removal of dicarbonyl metabolites, including MG, the most reactive and potentially dangerous dicarbonyl compound with hormetic potential [55,56]. MG mainly forms as a common by-product of glycolysis, including the spontaneous dephosphorylation of glyceraldehyde-3-phosphate (G3P) and dihydroxyacetone phosphate (DHAP), but also from the metabolism of lipids and proteins [33,57]. Due to its high reactivity, MG rapidly reacts with proteins, lipids, and nucleic acids, generating advanced glycation end products (AGEs), which in turn may generate cell and tissue dysfunctions in aging and disease. In particular, reaction with proteins is directed mainly to arginine residues, forming specific MG-adducts, such as 5-hydro-5-methylimidazolone (MG-H1) and argpyrimidine, the former representing one of the most quantitatively and functionally important AGEs in physiological systems [34]. Examples of molecular and cellular dysfunctions mediated by MG glycation of arginine residues are: (1) increased formation of reactive oxygen species (ROS) [58,59], (2) activation of inflammatory pathways mediated by the receptor for AGEs (RAGE) [60,61], (3) activation of the mitochondrial pathway of apoptosis [59,62,63,64,65,66], and (4) induction of epithelial-to-mesenchymal transition (EMT) [67,68,69]. Due to all these biochemical effects, AGEs have been associated with the pathogenesis of a number of diseases, including cancer [62,69], infertility [66,70,71,72,73], osteoporosis [59,74], obesity [75], diabetes and diabetic vascular complications, chronic renal disease, cardiovascular disease, and neurological diseases [34].

**Figure 1:**
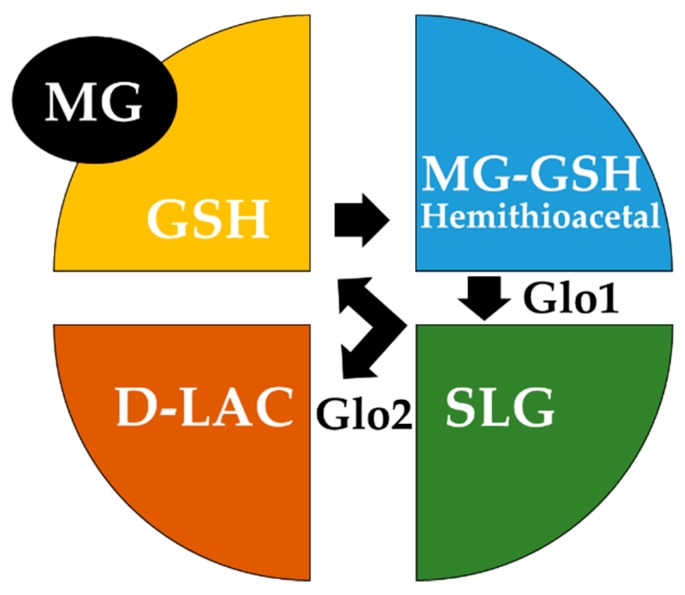
Schematic model of the glyoxalase system. The glyoxalase system consists of two glyoxalase enzymes, Glyoxalase 1 (Glo1) and Glyoxalase 2 (Glo2), and a catalytic amount of glutathione (GSH). Glo1 converts the hemithioacetal spontaneously formed between methylglyoxal (MG) and GSH to S-D-lactoylglutathione (SLG), whereas Glo2 catalyses the hydrolysis of SLG to D-lactate (D-LAC), regenerating GSH.

Under normal conditions, MG is metabolized by the enzyme Glyoxalase 1 (Glo1), using GSH as a cofactor [55]. To rapidly eliminate MG, Glo1 works in tandem with another enzyme, namely Glyoxalase 2 (Glo2). These enzymes catalyze the conversion of MG to D-lactate (D-LAC) via the so-called GSH-dependent glyoxalase pathway. Specifically, Glo1 converts the MG-GSH hemithioacetal, resulting from the nonenzymatic condensation of MG with GSH to S-D-lactoylglutathione (SLG), whereas Glo2 hydrolyses SLG, thus generating D-LAC and regenerating GSH [56] (Figure 1).

A mitochondrial D-LAC dehydrogenase is present in mammals and converts D-LAC into pyruvate, which is then rapidly reduced into L-lactate [56]. Certainly, due to this essential task in removing MG and MG-derived AGEs, the glyoxalase system plays also a pivotal role in controlling several pathological conditions, such as obesity [76], infertility [66,71], cancer [68,69,77,78,79,80], neurodegenerative diseases [81,82], diabetes, and cardiovascular diseases [34,83].

## 4. Role of Dicarbonyl Stress in Vascular Dysfunctions and Diseases

Accumulated experimental results have shown that dicarbonyl stress contributes significantly to endothelial cell dysfunction and impairment of both microvessels and large arteries, leading eventually to vascular diseases [33]. Specifically, there is evidence that MG can trigger human aortic endothelial cell dysfunction via modulation of ATP-sensitive potassium channels (K_ATP_) and the Ras/MAPK (mitogen-activated protein kinase) pathway [84]. Additionally, MG has been shown to induce aberrant angiogenetic responses that affect the stability and permeability of the endothelial barrier [35,36,37] and are clearly implicated in cardiovascular diseases associated with OS and inflammation [85,86,87]. Consistently, MG-induced carbonyl stress has been suggested to contribute to endothelial dysfunction through either activation of proinflammatory responses [88,89], inhibition of protective thiol-disulfide oxidoreductase systems [90], or apoptosis [91]. Moreover, it has been reported that MG may disrupt retinal vessel integrity, leading to hyperpermeability of the blood-retinal barrier [92], and alter vascular tone [93,94]. Furthermore, MG-derived AGEs, including MG-H1, were found in carotid atherosclerotic plaques associated with a rupture-prone phenotype [95]. Regarding more specifically the brain vasculature, substantial research has demonstrated that the strong and deleterious glycating power of MG has also a significant impact on cerebral vessels and cerebrovascular diseases. In an animal model of diabetes, elevated MG levels produced by vascular smooth muscle cells were indeed shown to impair endothelial cell-mediated vasodilatation of cerebral microvessels, eventually leading to cerebral ischemia [96]. Moreover, MG has been shown to cause OS-mediated injury on cultured human brain microvascular endothelial cells (hBMEC) through activation of the AGE/RAGE pathway [97], as well as to increase the production of ROS in human cerebral microvascular endothelial cells (hCMEC) via ERK/JNK-signaling and markedly reduce the integrity of the blood-brain barrier (BBB) [98]. Besides their detrimental effects, MG-derived post-translational modifications can also stimulate the activity of major stress-inducible proteins implicated in cell recovery from damage caused by stressful events, including OS and inflammation, and protection from apoptosis, thus playing a significant role in cell survival [48,99,100]. Consistently, there is also evidence that some MG-derived AGEs, including argpyrimidine, have significant antioxidant properties [101], as well as that MG is able to trigger autophagy in human brain microvascular endothelial cells as a feedback-defense mechanism against the injuries it can cause [102].

By controlling the intracellular levels of MG and, consequently, MG-derived AGEs, also Glo1 has been shown to play major and complex roles in vascular physiology and pathophysiology [103,104], including the modulation of brain microvascular endothelial barrier functions [105]. In particular, it has been reported that Glo1 decreases glycative and oxidative stress and limits the age-related decline in endothelial function by either modulating endothelial nitric oxide synthase (eNOS) phosphorylation [106] or hampering occludin glycation [107], as well as that these adaptive defense responses against glycative stress may be under the control of SIRT1 [108]. Conversely, there is evidence that reduction of Glo1 aggravates cerebrovascular remodeling by either promoting the proliferation of basilar smooth muscle cells in hypertension [109] or causing direct endothelial cell damage [110] and constitutes a common feature of endothelial and vascular dysfunctions [111]. Consistently, the excessive accumulation of MG and AGEs induced by Glo1 knockdown in mouse aortic endothelial cells has recently been shown to impair angiogenesis [112]. Finally, a study in human arterial tissues reported significantly lower Glo1 activity in atherosclerotic lesions, compared with normal tissue sections of the same blood vessels, suggesting that the depletion of Glo1 functionality contributes to an increased risk of cardiovascular disease [113,114]. On the other hand, Glo1 overexpression has been shown to prevent endothelial damage induced by MG-derived AGEs [115,116,117], reverse defective neovascularization in diabetic mice [118], reduce endothelial dysfunction in a rat model of diabetes [119], and favor angiogenesis [120]. Finally, increased Glo1 activity can improve arterial dilatation and decrease vascular inflammation [121].

Overall, these and other accumulated evidences demonstrate that dicarbonyl stress resulting from Glo1 failure to counteract MG dicarbonyl accumulation contributes significantly to the alteration of endothelial homeostasis and function that underlie the development of vascular diseases.

## 5. Role of Dicarbonyl Stress in CCM Disease

The role of dicarbonyl stress in CCM disease has been so far scarcely investigated. Using previously established cellular models of CCM disease, our group has recently demonstrated for the first time that KRIT1 loss-of-function induces a redox-sensitive upregulation of Glo1, which in turn causes a reduction in intracellular levels of MG-modified cytoprotective chaperone proteins, including heat-shock proteins 70 (Hsp70) and 27 (Hsp27), leading eventually to an increased cell susceptibility to oxidative DNA damage and apoptosis [48]. These results provided useful insights into CCM pathogenesis and the development of novel preventive and therapeutic strategies, as no direct therapeutic approaches for CCM disease exist so far besides surgical removal of accessible lesions [122]. In particular, our pioneering work demonstrated that the upregulation of Glo1 expression detected in KRIT1-knockout MEF cells and KRIT1-silenced human brain microvascular endothelial cells (hBMEC), two established models of CCM disease, is paralleled by a decrease in the intracellular levels of argpyrimidine (AP), a specific MG-derived protein glycation adduct that plays a dual, context-dependent role in the apoptotic process, acting as either a pro-apoptotic [64,65] or anti-apoptotic factor [99,100]. Specifically, we detected two major AP-modified proteins with approximate molecular weights of 70 and 27 kDa, which were subsequently identified as mouse Hsp70 and Hsp27; thus demonstrating that KRIT1 loss-of-function is associated with reduced intracellular levels of the AP-modified forms of two major heat-shock proteins known to exert protection against several stress stimuli in mammalian cells. More intriguingly, given that the AP-mediated post-translational modification of Hsp70 and Hsp27 has been demonstrated to endow these proteins with enhanced anti-apoptotic properties [99,100], our findings suggested a potential effect on cell susceptibility to apoptosis. Consistently, we found that the observed decrease in AP-modified Hsp70 and Hsp27 levels caused by KRIT1 loss-of-function was associated with increased levels of oxidative DNA damage and activation of the intrinsic (mitochondrial) pathway of apoptosis, which is especially susceptible to ROS [48]. Furthermore, by treating cells with the antioxidant tiron, a SOD mimetic mitochondria-permeable ROS scavenger, we demonstrated that the correlated upregulation of Glo1 and depletion of AP-modified Hsp70 and Hsp27 observed in KRIT1-null cells were a consequence of the increase in intracellular ROS levels caused by KRIT1 loss-of-function, thus further supporting and extending its redox-sensitive pleiotropic effects [41,42,44,47]. In addition, we found that KRIT1 loss-dependent upregulation of Glo1 is part of a cell adaptive response to OS involving the transcriptional factor Nrf2, a master regulator of antioxidant responses. In fact, KRIT1 loss-of-function leads to a persistent redox-dependent activation of Nrf2 and, in consequence, to a sustained upregulation of Glo1 [48]. Importantly, again, these results were confirmed in KRIT1-silenced hBMEC cells, demonstrating that KRIT1 downregulation causes a redox-dependent upregulation of Glo1 and subsequent depletion of AP-modified Hsp70 and Hsp27 in distinct cellular models of CCM disease. Finally, we demonstrated that these effects were also linked to defective autophagy and redox-dependent activation of JNK. Remarkably, we found that the upregulation of Nrf2, Glo1, and phosphorylated JNK (p-JNK) caused by KRIT1 loss-of-function occurs also in vivo, as shown by IHC analyses of paraffin-embedded surgically resected CCM specimens [48], suggesting that these molecular events may contribute to CCM disease pathogenesis. Consistent with our findings, while it is well-established that the Nrf2 pathway acts in concert with autophagy to counteract the deleterious effects of redox-damaged proteins, growing evidence demonstrates that the sustained activation of Nrf2 can affect essential oxidative post-translational modifications (OPTMs) of proteins involved in normal cell metabolism and signaling [123].

Taken together, these results provided novel and interesting insights into the potential role of Glo1/MG-dependent pathways in CCM disease, opening new and interesting avenues of investigation towards greater knowledge of the complex molecular puzzle involving KRIT1 and the pleiotropic effects of its dysfunction.

## 6. GSH and Protein S-Glutathionylation

The tripeptide GSH is found at high concentration in virtually all mammalian cells and constitutes one of the most versatile and potent biological nucleophiles, thereby playing critical roles in maintaining intracellular redox homeostasis and protecting cells from oxidative damage and the toxicity of xenobiotic electrophiles [124]. As the main intracellular thiol-based antioxidant compound, GSH can directly scavenge reactive oxygen/nitrogenspecies (ROS/RNS) or may act as a thiol cofactor for distinct GSH-dependent enzymes, including Glo1 and Glo2, glutathione reductase (GSR), glutaredoxins (GRXs), glutathione peroxidases (GPXs), peroxiredoxins (PRDXs), and glutathione transferases (GSTs) [55,125]. GSH is synthesized and mostly distributed in the cytoplasm, from where it can reach various organelles (nucleus, peroxisomes, mitochondria, and endoplasmic reticulum) through specific transporters [126,127]. Major enzymes involved in the synthesis of GSH are glutamate cysteine ligase (GCL) and glutathione synthetase (GS), while γ-glutamyl transferase (GGT) is the enzyme that catabolizes GSH [128]. In physiological conditions, intracellular GSH exists mainly in the reduced monomeric form (GSH), whereas the disulfide dimer deriving from its oxidation (GSSG) is present at a much lower concentration. Indeed, the molar ratio between these two reversible forms of GSH (GSH:GSSG) exceeds 100:1 in a resting cell, representing the major cellular redox buffer, while it shifts in favor of GSSG during aging and can decrease to values of 10:1 and even 1:1 as a consequence of prolonged OS, thus serving as a marker of such stressful conditions [125,129]. Despite its well-established role as an antioxidant, GSH has also been shown to induce pro-oxidant effects, including lipid peroxidation, due to the removal of the γ-glutamate residue from the cysteine residue that occurs during its catabolism, eventually leading to the activation of intracellular signaling pathways and enhanced production of ROS and free radicals that may further increase lipid peroxidation and cause DNA damage [130,131]. Consistently, there is evidence that the pro-oxidant effect of GSH can play a role in vascular injury and atherogenesis by enhancing the oxidation of low-density lipoproteins (LDL) [132]. On the other hand, it has been shown that GSH may form an S-nitroso-glutathione (GSNO) adduct by conjugating to nitric oxide (NO). In turn, the GSNO adduct, which represents one of the major transport forms of NO in biological systems, may exert a protective effect during cell exposure to oxidants through transnitrosation and S-thiolation reactions. S-nitrosoglutathione reductase (GSNOR) catalyzes the breaking of GSNO, thus modulating the NO bioavailability. The dysregulation of GSNOR can lead to drastic changes in protein S-nitrosylation, with pathological consequences [133]. In addition, it has been demonstrated that GSH is implicated in many other cellular functions, including proliferation, apoptosis, cell cycle control, catabolism of xenobiotics and lipid and deoxyribonucleotide metabolism, as well as S-glutathionylation of proteins.

S-glutathionylation is a reversible oxidative post-translational modification (OPTM) of proteins characterized by the formation of mixed disulfides between the thiol of GSH and a thiol group of a target protein [134]. Consequently, it represents an important regulatory mechanism that controls the activity of different proteins, including target proteins with regulatory functions in redox signaling [135], and has clinical implications in numerous human diseases, including diabetes, cancer, cardiovascular, and neurodegenerative diseases [136]. The formation of protein-SSG mixed disulfides occurs often under mild oxidative/nitrosative stress as an adaptive cellular response to prevent irreversible oxidation of protein thiols and may be associated with pathological events [137]. However, it plays also a fundamental role under basal physiological conditions as a biological redox switch in the regulation of signaling and metabolic pathways [135]. In particular, a number of mitochondrial proteins have been shown to be highly susceptible to reversible S-glutathionylation, including proteins involved in mitochondria fission and fusion, energy metabolism, solute transport, ROS production, antioxidant defense, and apoptosis [138,139]. Moreover, there is evidence that S-glutathionylation can reversibly reduce the activity of endothelial nitric oxide synthase (eNOS), leading to loss of NO and gain of O_2_•^–^ and consequent vascular effects [32]. Regarding the mechanisms of protein S-glutathionylation, it has been reported that protein thiol moieties might be converted to protein-SSG mixed disulide adducts via either thiol-disulfide exchange or various reactive intermediates formed under OS conditions [136]. Furthermore, distinct enzymes have been implicated in catalysis of S-glutathionylation and deglutathionylation reactions, including glutathione-S-transferase (GST) and glutaredoxin (GRX) [136]. In addition, a possible role for the Glo2 enzyme in S-glutathionylation of specific proteins has been also reported [140,141].

Overall, the very close relationship between GSH-dependent redox homeostasis and protein S-glutathionylation is now a matter of fact and has been extensively reported in previous reviews [136,142]. Indeed, taking into account the multifaceted aspects of this functional relationship, it is not surprising that its alteration plays an important etiological role in the onset and progression of numerous dysfunctions and diseases, including endothelial dysfunction [143] and associated cardiovascular diseases [31,132,144,145,146].

## 7. GSH and S-Glutathionylation in Vascular Dysfunction and Diseases

In general, GSH plays an important protective role in the pathogenesis of vascular diseases by counteracting the detrimental effects exerted by OS and inflammation [132,147,148]. Dysregulation of the two cytosolic enzymes of GSH synthesis, including γ-glutamylcysteine synthetase (GCS) and GSH synthase (GS) and the various GSH-dependent enzymes, was observed in endothelial dysfunction [32]. One of the proposed models of how an altered redox homeostasis may contribute to vascular endothelial dysfunction and diseases is based on the fact that a low GSH:GSSG intracellular ratio results in a diminished capacity to scavenge ROS and RNS. In turn, the oxidative burden favors S-glutathionylation (oxidation) of cysteine residues 689 and 908 in eNOS, leading to uncoupled eNOS and the consequent reduced synthesis of NO and increased production of superoxide anions (O_2_•^–^). The O_2_•^–^ overproduction may then result in multiple detrimental effects, including the formation of the highly reactive intermediate peroxynitrite (ONOO^−^) and generation of RNS through the facilitated reaction between O_2_•^–^ and NO, eventually culminating in oxidative damage to proteins, lipids, and DNA and vascular endothelial dysfunction and diseases [32]. Indeed, whereas depressed GSH synthesis has been shown to precede OS and atherogenesis in apolipoprotein E-deficient mice [149], the S-glutathionylation of endothelial cell proteins has emerged as a novel stress-induced redox mechanism that may underlie vascular barrier dysfunctions and diseases [150,151]. Accordingly, it is well-established that the redox state of cysteine residues is critical for the function of redox-responsive proteins involved in endothelial cell homeostasis and functions [152]. Furthermore, there is evidence that physiological levels of GSH have antioxidant and anti-atherogenic properties and may lead to remission of atherosclerosis [153,154]. In particular, it has been reported that increasing GSH levels by administration of N-acetyl-cysteine (NAC), a GSH precursor that has been used in therapeutic practices [155], leads to a downregulation of the proinflammatory vascular cell adhesion protein 1 (VCAM-1), a pathogenic factor in atherosclerosis, and prevents vascular damage in patients with diabetes [156].

In addition, the intimate relationship between OS and altered protein S-glutathionylation has been shown to have a significant impact on aortic valve sclerosis (AVS), an atherosclerosis-associated vascular disease characterized by increased carotid intima-media thickness, carotid and coronary plaques, and altered flow-mediated dilation [29,157]. In this context, endothelial dysfunction represents the initial trigger of aortic leaflet structural deterioration and has been attributed to the alteration of GSH homeostasis in endothelial cells. Indeed, altered GSH homeostasis may contribute to both the reduction of antioxidant defences and the abnormal increase in protein S-glutathionylation that have been asscociated with oxidative DNA damage and endothelial-to-mesenchymal transition (EndMT). Hence, restoring GSH homeostasis may represent a strategy to counteract endothelial dysfunction and vascular diseases [29]. Nevertheless, it should not be neglected that the growing evidence of significant harmful effects of reductive stress in the cardiovascular system, including the paradoxical capacity of excessive reducing equivalents to stimulate mitochondrial ROS production and OS and promote injury, thereby highlights the complexity of developing therapies that affect the intricately connected redox states of biological systems [123,158,159,160].

## 8. Role of GSH and S-Glutathionylation in CCM Disease

Although the GSH redox system has been clearly implicated in the physiology of cerebral microvascular endothelial cells and shown to play a protective role against oxidative disruption of the endothelial barrier function and development of neurovascular disorders induced by OS [161,162], the role of this system and the associated S-glutathionylation in the pathogenesis of an important cerebrovascular disease, such as CCM disease, has been investigated only recently by our group [51]. Using previously established cellular models of CCM disease, including KRIT1-knockout mouse embryonic fibroblasts (MEFs) and KRIT1-silenced hBMECs, we found that KRIT1 loss-of-function affects the GSH redox system, leading to a significant decrease in intracellular GSH levels and GSH:GSSG redox ratio and an increase in the oxidized GSSG disulfide form, which overall reduced the intracellular antioxidant capacity. Furthermore, we showed that the decreased GSH:GSSG ratio and increased intracellular oxidation state were associated with an enhanced S-glutathionylation of various proteins in KRIT1-null vs. KRIT1-expressing cells. In particular, redox proteomic analysis by nLC-ESI-LIT-MS/MS identified distinct proteins that were differentially S-glutathionylated as a function of KRIT1 expression, including important cytoskeletal proteins, such as actin; tubulin beta-4B chain; tropomyosin; and vimentin; the glycolytic enzymes glyceraldehyde-3-phosphate dehydrogenase (GAPDH) and alpha-enolase (ENO1), creatine kinase B-type (CKB); and members of the chaperonin family, such as heat shock protein 60 (HSP60) and calreticulin (CALR). Notably, all the identified S-glutathionylated proteins were known to be redox-sensitive and implicated in fundamental biological processes linked to cellular adaptive responses to OS, including the unfolded protein response (UPR), the interplay between oxidant species and energy metabolism, and the cytoskeleton organization and dynamics, as well as in the maintenance of BBB integrity and functionality [163,164,165]. In particular, given that a coordinated dynamics of actin filaments and microtubules is crucial for the regulation of endothelial barrier stability and vascular permeability [166], it is significant that such dynamics are redox-sensitive and may be modulated by S-glutathionylation of major structural and regulatory proteins [15,167]. Importantly, IHC analysis of distinct CCM surgical specimens from KRIT1 loss-of-function mutation carriers demonstrated that an increased accumulation of S-glutathionylated proteins occurs also in endothelial cells lining the lumen of abnormally dilated CCM vessels, compared with perilesional normal vessels, thus providing clinical relevance to the in vitro results and suggesting a potential correlation with CCM disease progression and severity [51].

Overall, these findings provided strong evidence that a reduction in the GSH:GSSG redox ratio caused by KRIT1 loss-of-function mutations results in enhanced S-glutathionylation of distinct structural and regulatory proteins, thus revealing a novel molecular signature in the stunning complexity of CCM disease and providing a novel framework for the identification of new disease biomarkers and therapeutic targets and the designing of specific and efficacious pharmacologic interventions.

## 9. Concluding Remarks

**Figure 2:**
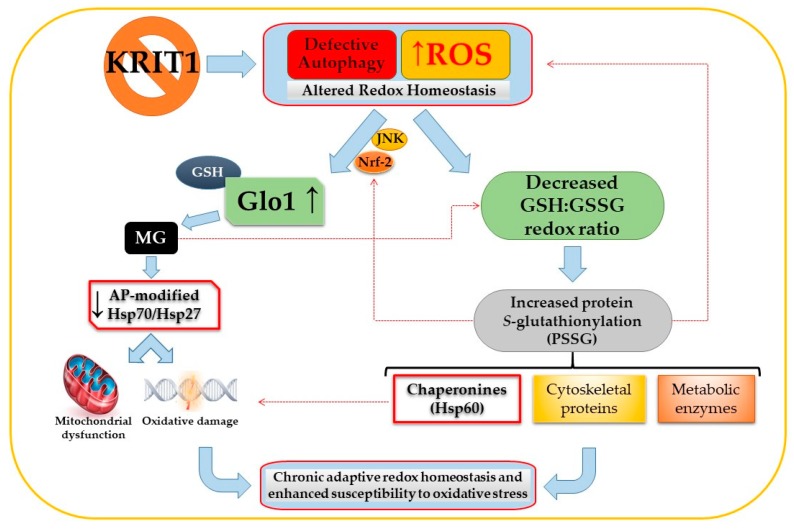
Potential interplay between methylglyoxal (MG)-mediated glycation and S-glutathionylation pathways induced by KRIT1 (*Krev interaction trapped 1*) loss-of-function. The impairement of intracellular redox homeostasis caused by KRIT1 loss-of-function leads to a reactive oxygen species (ROS)-dependent sustained activation of the JNK-Nrf2 (c-Jun N-terminal kinase -nuclear factor erythroid 2-related factor) pathway and upregulation of downstram targets, including Glyoxalase 1 (Glo1). In turn, Glo1 upregulation results in decreased intracellular levels of cytoprotective MG adducts, including argpyrimidine (AP)-modified heat-shock proteins (HSP) 27 and 70, leading to an increased cell susceptibility to oxidative damage and mitochondria-dependent apoptosis. Concomitantly, KRIT1 loss-of-function affects the glutathione (GSH) redox system, causing a significant decrease in the GSH:GSSG redox ratio and an increase in the S-glutathionylation of important structural and regulatory proteins, including metabolic enzymes; cytoskeletal proteins; and chaperonines, such as the HSP60. A potential interplay between the MG-mediated glycation and S-glutathionylation pathways may also occur, including the modulation of the GSH:GSSG redox ratio by MG and the contribution of S-glutathionylation to ROS production and Nrf2 activation (hatched red lines), leading to a synergistic contribution to the chronic adaptive redox homeostasis and enhanced cell susceptibility to oxidative stress associated with KRIT1 loss-of-function mutations. Eventually, these synergistic pathological effects might therefore culminate in cerebral cavernous malformation (CCM) disease onset and severity. See text for details.

A common feature in glycative and oxidative post-translational modifications of proteins, such as MG-mediated glycation and S-glutathionylation, is the modulation of protein biological activity and stability [168,169,170,171]. Remarkably, in cellular models of CCM disease, these modifcations affect proteins involved in cellular homeostasis and adaptive responses to stressful conditions, including prominent members of the heat-shock protein (HSP) family functioning as molecular chaperones, such as HSP70, HSP27, and HSP60; enzymes of energy metabolism; and cytoskeleton proteins, suggesting that they play an important role in the chronic adaptive redox homeostasis and enhanced cell susceptibility to OS and inflammation that have been associated with loss-of-function mutations of CCM genes [48]. In this light, it is plausible that MG-mediated glycation and S-glutathionylation of distinct target proteins involved in cellular stress responses, and consequent downstream effects, including endothelial cell dysfunction, may occur simultaneously, thus raising the possibility that these two mechanisms of protein post-translational modifications can influence the pathogenesis of CCM disease in a synergistic manner. Consistently, there is evidence that both mechanisms are affected by KRIT1 loss-of-function in the same cellular models and surgical samples of CCM disease, as well as that they are significantly implicated in distinct vascular dysfunctions and diseases. In addition, it is also possible to glimpse potential upstream regulatory mechanisms that could orchestrate a putative molecular crosstalk between MG-mediated glycation and S-glutathionylation and coordinate their downstream effects. Indeed, both mechanisms have been shown to be influenced by the abnormal alteration of cellular redox homeostasis that occurs upon KRIT1 loss-of-function [48,49,51] (Figure 2). 

Moreover, given the evidence that MG can cause dysfunction of thiol-disulfide oxidoreductase systems that protect endothelial cells against OS, including the thioredoxin/thioredoxin reductase system [90], it is also possible to hypothesize that dicarbonyl stress may decrease the GSH:GSSG ratio and, thereby, stimulate S-glutathionylation. Conversely, there is evidence that S-glutathionylation may contribute to ROS production and altered redox homeostasis by affecting the activity of eNOS [32]. Furthermore, it has been reported that S-glutathionylation of Keap1, an endogenous repressor of Nrf2, can be implicated in the activation of Nrf2 [172], suggesting a potential mechanistic contribution to the sustained activation of this antioxidant transcription factor that occurs upon KRIT1 loss-of-function and, in consequence, to the upregulation of Nrf2 downstream targets, including Glo1. Overall, these potential mechanisms of molecular crosstalk bethween the ROS/Nrf2/Glo1/MG-mediated glycation and the S-glutathionylation pathways might contribute to the chronic adaptive redox homeostasis and enhanced cell susceptibility to OS associated with KRIT1 loss-of-function mutations [48,49].

While the specific effects of glycative and oxidative post-translational modifications of proteins triggered by loss-of-function mutations of KRIT1, including MG-dependent glycation and S-glutathionylation of important structural and regulatory proteins, remain to be defined, the comprehensive characterization of their upstream regulatory mechanisms and functional interplay should provide novel insights into CCM disease pathogenesis and enable the development of targeted, safe, and effective synergistic drug combination therapies.

Future studies could fruitfully explore this emerging and intriguing scenario.
